# Serum CRP interacts with SPARC and regulate immune response in severe cases of COVID-19 infection

**DOI:** 10.3389/fimmu.2023.1259381

**Published:** 2023-11-23

**Authors:** Chengyang Liu, Chenyang Zheng, Xipeng Shen, Ling Liang, Qiuyu Li

**Affiliations:** ^1^ Department of Pathology, Institute of Systems Biomedicine, School of Basic Medical Sciences, Peking-Tsinghua Center for Life Sciences, Beijing Key Laboratory of Tumor Systems Biology, Peking University Health Science Center, Beijing, China; ^2^ School of Biological Science and Medical Engineering, Beihang University, Beijing, China; ^3^ Department of Biophysics, School of Basic Medical Sciences, Peking University Health Science Center, Beijing, China; ^4^ Department of Respiratory and Critical Care Medicine, Peking University Third Hospital, Beijing, China

**Keywords:** C-reactive protein, COVID-19, secreted protein acidic and rich in cysteine, cytokine response storm, megakaryocyte

## Abstract

Serum C-reactive protein (CRP) has been found elevated during COVID-19 infection, and associated with systematic inflammation as well as a poor clinical outcome. However, how did CRP participated in the COVID-19 pathogenesis remains poorly understood. Here, we report that serum C-reactive protein (CRP) levels are correlated with megakaryocyte marker genes and could regulate immune response through interaction with megakaryocytes. Molecular dynamics simulation through ColabFold showed a reliable interaction between monomeric form of CRP (mCRP) and the secreted protein acidic and rich in cysteine (SPARC). The interaction does not affect the physiological activities of SPARC while would be disturbed by pentamerization of CRP. Interplay between SPARC and mCRP results in a more intense immune response which may led to poor prognosis. This study highlights the complex interplay between inflammatory markers, megakaryocytes, and immune regulation in COVID-19 and sheds light on potential therapeutic targets.

## Introduction

One crucial feature of COVID-19 infection is the upregulation of serum C-reactive protein (CRP) level, an acute-phase protein usually considered as a sensitive index of tissue injury. CRP is classically synthesized in the liver hepatocytes upon interleukin-6 (IL-6) induction, consisting of five non-covalently linked subunits forming a disc-shaped pentamer (pCRP) and released to plasma circulating in pentameric form (1). At the inflammation and infection sites, CRP interacts with the bioactive lipids on the cell membrane of activated platelet or target cells and dissociates into monomeric subunits called mCRP. Retrospective studies show that both the native pCRP and mCRP have predictive value of clinical severity in COVID-19 disease (2, 3).

Mechanically speaking, CRP participates in innate immunity through interaction with C1q and consequent activation of the complement pathway or binding to Fc receptors with the resulting release of pro-inflammatory cytokines (4). Previous clinical evidence suggests that CRP is elevated in bacterial rather than viral infections and usually lacks adaptive immunity. The high serum CRP levels during COVID-19 infection are associated with cytokine response storm (hypercytokinemia) or macrophage activation syndrome (2). Nevertheless, the impact of C-reactive protein (CRP) on the progression of COVID-19-associated pneumonia remains to be elucidated. Here, using open-access databases and clinical retrospective studies, we proposed a model of how CRP regulates immune responses in COVID-19 infection.

## Methods

### Collection of CRP-associated genes in COVID 19 patients

We selected whole blood RNA-seq datasets of COVID-19 patients from the Gene Expression Omnibus (GEO) database (https://www.ncbi.nlm.nih.gov/geo/) for discovery and validation of CRP-associated transcripts. Dataset (GSE157103) were used for discovery research (5). By performing Pearson correlation analysis, we obtained five transcripts that was positively correlated with serum CRP level. Dataset GSE172114, GSE167930 were used for verification (6, 7). Dataset GSE158055 were used for single-cell level analysis, http://covid19.cancer-pku.cn (8). This study was reviewed by the Ethics Committee of Peking University Third Hospital (IRB00006761-M2020060).

### Protein–protein interaction analysis and network construction

We constructed a PPI network using common transcriptes and employed STRING (9), setting a minimum required interaction score of 0.4, while keeping other parameters at their default values. Subsequently, we analyzed and visualized the PPI results using Cytoscape (version 3.10.0) (10). To identify key proteins within the network, we utilized cytoHubba, a Cytoscape plug-in, and applied the degree topological algorithm to obtain the five hub proteins with the highest degree values.

### Molecular dynamics (MD) simulation analysis

The three-dimensional protein complex structure of mCRP interacting with SPARC was predicted using AlphaFold2 (11) as implemented in ColabFold (12) running locally in the alphafold2_multimer_v3 model. The Amber-relaxed, top-ranked AlphaFold2 structure was used for MD simulation. The CHARMM-GUI website is used to process the protein file, and 150 mM NaCl was added to mimic physiological conditions. Prepared systems were first minimized using 5000 steps of a steepest descent algorithm. Next, 125 ps was used to equilibrate the system at 310 K, and a 300 ns MD simulation was conducted at a constant temperature of 310 K using the Gromacs 2023 software package. VMD was used to process and analyze the protein structure. The interface was analyzed by PISA available at https://www.ebi.ac.uk/pdbe/pisa/ and the results were displayed using Pymol (The PyMOL Molecular Graphics System, Version 2.0 Schrödinger, LLC).

### Gene ontology and pathway enrichment analyses

In order to gain insights into the functional characteristics of CRP-associated megakaryocyte marker genes, a set of enrichment analyses was performed using R package Enrichr (13). This approach aimed to provide detailed information on the biological mechanisms and signaling pathways associated with these genes. The enrichment analyses encompassed gene ontology (GO) terms, including biological process, molecular function, and cellular component categories. Additionally, to achieve a more comprehensive understanding of the relevant signaling pathways, other databases such as WikiPathways, Reactome, BioCarta, and the KEGG pathway were also employed in the analysis.

## Results

### Serum CRP level is highly correlated with megakaryocyte marker genes during COVID-19 infection

**Figure 1:**
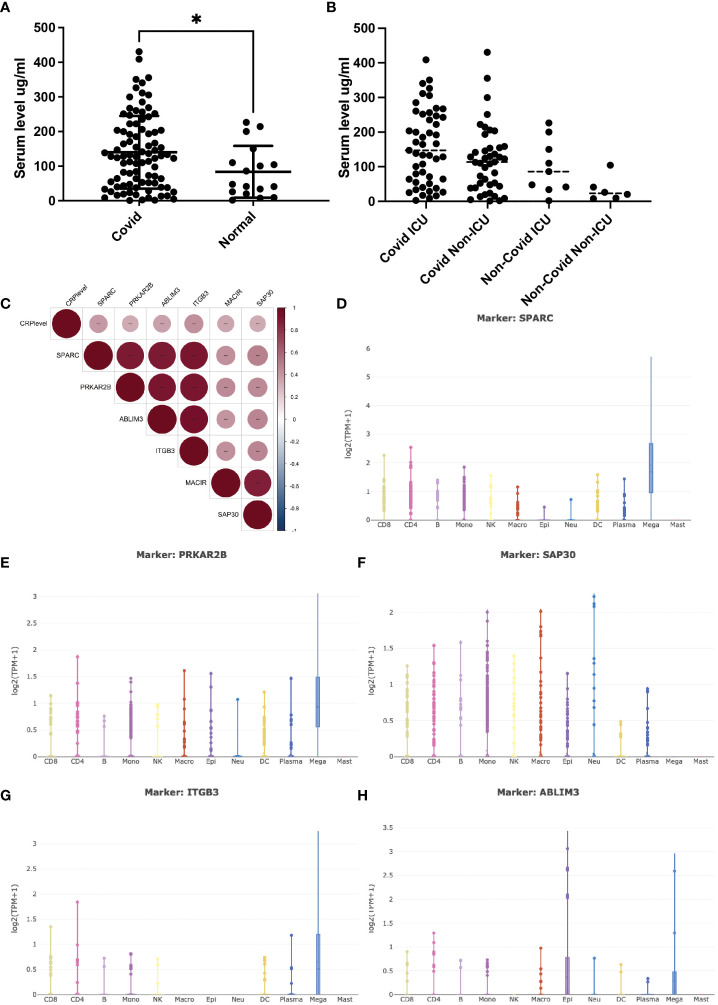
Serum CRP level and its related transcripts during Covid-19 infection. **(A)** (A) Serum CRP level (ng/ml) in Covid-19 patients collected from Peking university third hospital. Two tailed unpaired t-test was performed, p=0.0362. *: p<0.05. **(B)** Serum CRP level (ng/ml) in GSE157103 dataset; **(C)** Pearson correlation analysis of CRP-associated transcripts in GSE157103 dataset; **(D–H)** Single-cell analysis of CRP-associated transcripts in major blood celltypes.

The serum C-reactive protein (CRP) level has been identified as a marker correlating with the severity of COVID-19 infection. Higher CRP levels often indicate a more pronounced inflammatory response, leading to widespread inflammation in the body, causing tissue damage, organ dysfunction, and poorer clinical outcomes (14). Clinical studies of COVID-19 patients were integrated, and the expression trend of serum CRP during the disease process was depicted (**Figures 1A, B**). Collectively, patients bearing COVID-19 infection exhibited significantly higher levels of serum CRP (>100mg/ml) compared to either the healthy ones or non-COVID patients with respiratory infection. Furthermore, patients with severe COVID-19 infection, often with critical pneumonia and systemic symptoms, showed high CRP levels, probably due to inflammatory factor storm. This gradual upward trend aroused our curiosity about whether CRP contributed to the disease progression and, if so, how CRP worked.

Open-access RNA-seq databases of COVID-19 patients were mined to illustrate the specific interactions and mechanisms by which CRP contributed to COVID-19 infection (5). Pearson correlation analysis identified a group of 6 transcripts that were significantly positively associated with serum CRP level (**Figure 1C**). Of these transcripts, ITGB3 was one of the vital predictive genes of COVID-19-related stroke as involved in integrin pathway signal transduction (15). PRKAR2B was a cAMP-MAPK kinase, which may bind to the NSP13 protein of SARS-COV-2 (16). SAP30 was a subunit of the histone deacetylase complex, which regulates gene acetylation modification levels and gene expression by binding to the HDAC complex (17). ABLIM3 was a microfilament-binding protein localized to microfilament stress fibers. MACIR was a macrophage immunometabolism regulator that only showed up in one RNA-seq database. SPARC was a glycoprotein that regulated the extracellular matrix and had been reported to be a metabolic, immune checkpoint for inflammation and interferon responses, participating in the TGF-beta/TNF signaling pathway and converting anti-inflammatory macrophages into pro-inflammatory macrophages (18). The latter three had never appeared in COVID-19-related research.

To verify whether and how these six transcripts were involved in COVID-19 infection along with CPR, single-cell RNA-sequencing databases were used to depict their cell-type specific expression. As shown in **Figures 1D–H**, most of these transcripts were mainly expressed by megakaryocytes (MK), while SAP30 was universally expressed in lymphoid and myeloid cells. As the precursors of platelets, MKs undergo a complex process of maturation and fragmentation to produce platelets. Megakaryocytes controlled the proliferation of hematopoietic cells, promoted the excretion of neutrophils from the bone marrow, and were not typically associated with acute inflammatory disease. In COVID-19 patients, abnormalities in blood parameters such as lymphopenia (reduced lymphocyte count) and thrombocytopenia (reduced platelet count) have been observed. Several lines of evidence suggested that MKs were significantly accumulated in progression/severe COVID-19 as a feature of the systemic inflammatory response, with SPARC being the marker gene (19, 20). However, whether and how CRP contributed to this process remains unclear, and it is essential to elucidate the direct correlation between CRP and MKs.

### SPARC interacts with mCRP through its Kazal-like domain

**Figure 2:**
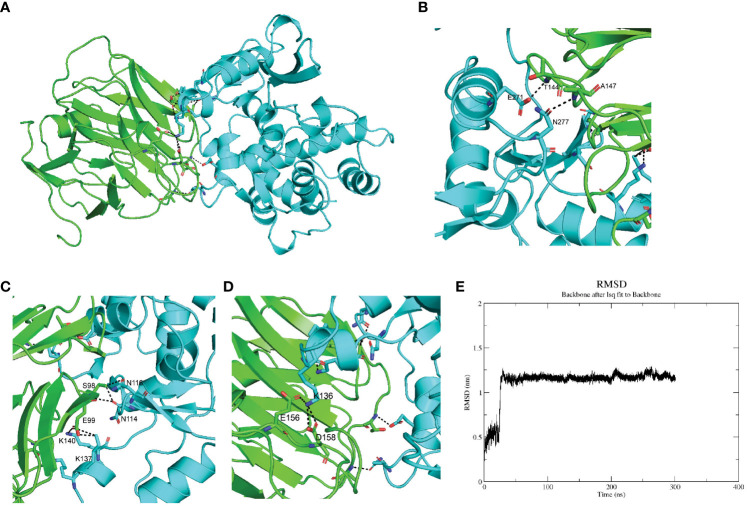
SPARC interacts with mCRP through its Kazal-like domain. **(A)** Molecular dynamics stimulation of mCRP-SPARC interaction models. green: mCRP; blue: SPARC. **(B–D)** Details of the interacting residues forming salt bridges and hydrogen bonds; **(E)** Root mean square deviation (RMSD) of mCRP-SPARC with respect to the initial structure during a 300 ns simulation.

Whether serum CRP interacted with the megakaryocytes’ signature genes was the first thing to be investigated. Since ABLIM3 and PRKAR2B were mainly expressed in the cytoplasm, we focused on the membrane protein ITGB3 and secreted protein SPARC. Furthermore, previous studies had suggested SPARC as a marker gene of MKs during cell type identification of scRNA-seq data (21, 22). Herein, ColabFold and molecular dynamics simulation were utilized to investigate SPARC-CRP interaction. Alphafold2 showed that mCRP exhibited a reliable interaction with SPARC (**Figure 2A**). Three clusters of intermolecular interactions were formed, as shown in **Figures 2B–E**, including five hydrogen bonds and three salt bridges. The distance between interacting residues was around 2-3 Å. The entire model stabilizes during 300ns in molecular dynamics (**Figure 2E**).

As a secreted protein, SPARC has been reported to share interaction with multiple receptors and cell surface matrix-associated molecules, like CPS1 and COL1A1 (23, 24). However, previous studies could have illustrated the exact amino acids responsible for these interactions. SPARC has a unique structure composed of an N-terminal acidic domain, a follistatin-like domain, a Kazal-like domain, and a C-terminal extracellular calcium-binding (ECB) domain (25). Through molecular dynamics, we could predict residues involved in interaction with mCRP. As shown in **Table 1**, most centered around the Kazal-like domain of SPARC, and the other two residues were in the ECB domain. Residues responsible for calcium binding (e.g., D222, P225, E227, Y229) and for collagen binding (e.g., N156, R164, E246) were not affected by SPARC-mCRP linking, suggesting that physiological functions of SPARC were not affected by this interaction (26, 27).

**Table 1:** Intermolecular interactions between SPARC and CRP.

Hydrogen bonds				Salt bridges		
	SPARC	Dist.[Å]	CRP	SPARC	Dist.[Å]	CRP
1	ASN 114[O]	3.51	GLU 99[N]	LYS 136[NZ]	2.60	GLU 156[OE1]
2	ASN 114[O]	2.55	SER 98[OG]	LYS 136[NZ]	2.69	ASP 158[OD1]
3	ASN 116[OD1]	3.31	SER 98[OG]	LYS 136[NZ]	3.32	ASP 158[OD2]
4	GLU 271[OE2]	3.10	THR 144[N]	LYS 137[NZ]	3.86	GLU 99[OE1]
5	ASN 277[OD1]	2.83	ALA 147[N]	LYS 137[NZ]	2.66	GLU 99[OE2]
6				LYS 140[NZ]	2.64	GLU 99[OE1]

The whole interface of SPARC-mCRP linking involved 54 residues collectively (**Table 2**). Residues of mCRP were mainly located on the beta-sheets (aa92-106, aa136-153), the structure of which remained stable and uniform in comparison with other peptides of mCRP. Aa35-47 and aa199-206 sequence of mCRP were reported to encompass potent ligand binding ability due to its soft, disordered conformation (28). However, none of these sequences form interaction with SPARC. Residues of mCRP on the interface were not fully investigated previously. Furthermore, in the pentameric form, the interface of mCRP was partially buried, indicating that the pentamerization of CRP would affect its binding to SPARC. Since pCRP dissociates into mCRP to promote inflammation, we proposed that the SPARC-mCRP linking may further aggravate inflammation status in COVID-19 patients.

**Table 2:** Interface summary.

		SPARC		CRP	
Number of atoms	interface	92	3.80%	97	4.90%
	surface	1509	63.10%	988	49.90%
	total	2391	100.00%	1981	100.00%
Number of residues	interface	23	9.20%	31	15.00%
	surface	246	98.80%	183	88.40%
	total	249	100.00%	207	100.00%
Solvent-accessible area, Å	interface	867.3	5.10%	804	7.70%
	total	17116.8	100.00%	10483.2	100.00%
Solvation energy, kcal/mol	isolated structure	-222.2	100.00%	-207.5	100.00%
	gain on complex formation	0.9	-0.40%	0.8	-0.40%
	average gain	-1.2	0.60%	-2	1.00%
	P-value	0.793		0.862	

### SPARC-mCRP linking affects inflammation status through megakaryocytes

**Figure 3:**
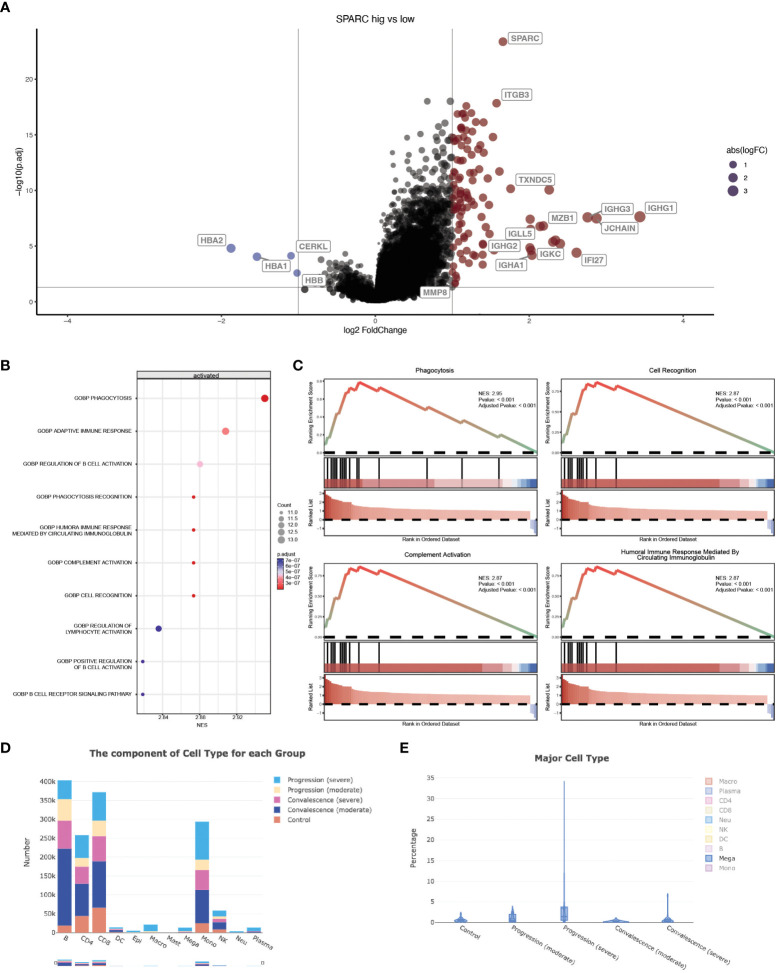
SPARC-mCRP link influence immune response in COVID-19 patients. **(A)** Volcano plot showing differentially expressed transcripts (DET), patients were clustered into two groups according to SPARC expression level, DET were identified through limma regression; **(B)** GSEA enrichment of DETs between clusters; **(C)** GSEA plot showing the most influenced pathway in SPARC-high vs low comparison; **(D, E)** Change of megakaryocytes proportion during different infection stages.

Previous studies have revealed that CRP could potentiate the IL-1-rich-microparticle production in megakaryocytes and further promote systemic inflammation (29, 30). Using SPARC as the primary classifier, patients were clustered into two groups (**Figure 3A**). The volcano plot showed that four transcripts were downregulated in SPARC-high vs low comparison, while 112 transcripts were upregulated. Gene Set Enrichment Analysis (GSEA) of the differentially expressed genes showed a more intense immune response in the SPARC-high group, characterized by overactivation of phagocytosis, adaptive immune response, B-cell activation, and complement activation pathways (**Figures 3B, C**). Notably, although the primary source of SPARC expression is megakaryocytes, no abnormalities in coagulation or platelet function were found.

Accumulation of megakaryocytes in severe progression cases and depression in convalescence patients was observed (**Figures 3D, E**; **Table 3**). Previous research identified an expansion of circulating megakaryocytes and increased erythropoiesis with features of hypoxic signaling in critical patients. However, most of these studies concluded that the expansion of MK led to hypercoagulability and thrombophilia in patients. Few articles mentioned the impact of MK cells on immune status. Our research proposed the possibility of megakaryocytes influencing inflammation response during COVID-19 through the SPARC-mCRP link. Further studies will be needed to illustrate the detailed mechanism of how CRP drove the expansion of megakaryocytes and the role of megakaryocytes in Covid-19 progression.

**Table 3:** GSEA analysis of SPARC-high vs low comparison.

GOBP Pathway	Enrichment Score	NES	p.adjust
Activation of immune response	0.75	2.68	3.73E-06
Adaptive immune response	0.76	2.91	3.84E-07
Adaptive immune response based on somatic recombination of immune receptors built from immunoglobulin superfamily domains	0.75	2.68	3.73E-06
Antigen receptor mediated signaling pathway	0.75	2.68	3.73E-06
B cell activation	0.71	2.71	3.73E-06
B cell mediated immunity	0.82	2.81	8.74E-07
B cell receptor signaling pathway	0.82	2.82	7.36E-07
Biological process involved in interspecies interaction between organisms	0.52	2.33	0.0005447
Cell activation	0.51	2.26	0.00063476
Cell recognition	0.86	2.87	2.02E-07
Complement activation	0.86	2.87	2.02E-07
Defense response	0.52	2.32	0.00029081
Defense response to bacterium	0.66	2.61	3.73E-06
Defense response to other organism	0.57	2.49	9.85E-05
Endocytosis	0.69	2.67	7.21E-06
Humoral immune response	0.70	2.69	4.18E-06
Humoral immune response mediated by circulating immunoglobulin	0.86	2.87	2.02E-07
Immune effector process	0.73	2.73	2.71E-06
Immune response	0.56	2.52	5.30E-05
Immune response regulating cell surface receptor signaling pathway	0.75	2.68	3.73E-06
Immune response regulating signaling pathway	0.66	2.52	3.29E-05
Innate immune response	0.67	2.72	3.73E-06
Leukocyte mediated immunity	0.78	2.75	1.21E-06
Lymphocyte activation	0.63	2.52	2.01E-05
Lymphocyte mediated immunity	0.82	2.81	8.74E-07
Membrane invagination	0.78	2.76	1.04E-06
Membrane organization	0.72	2.64	4.38E-06
Phagocytosis	0.79	2.95	2.02E-07
Phagocytosis recognition	0.86	2.87	2.02E-07
Positive regulation of b cell activation	0.82	2.82	7.36E-07
Positive regulation of cell activation	0.75	2.70	3.73E-06
Positive regulation of immune response	0.75	2.68	3.73E-06
Positive regulation of immune system process	0.66	2.61	3.73E-06
Regulation of b cell activation	0.80	2.88	4.40E-07
Regulation of cell activation	0.64	2.56	1.44E-05
Regulation of immune response	0.66	2.52	3.29E-05
Regulation of immune system process	0.56	2.39	0.00036199
Regulation of lymphocyte activation	0.77	2.84	7.28E-07
Response to bacterium	0.66	2.61	3.73E-06
Vesicle mediated transport	0.68	2.72	2.71E-06

## Discussion

Our study aimed to investigate how CRP regulates the immune responses during COVID-19 infection. Using open-access databases and clinical retrospective studies, we noticed that serum CRP interacted with circulating megakaryocytes through SPARC and further regulated virus response and systematic inflammation in COVID-19 patients. We discovered a positive correlation between serum CRP levels and the proportion of circulating megakaryocytes.

The positive correlation between serum CRP levels and circulating megakaryocyte proportion suggests a potential link between inflammation and megakaryocyte biology. It is well-established that CRP is an acute-phase reactant produced by the liver in response to inflammation. Elevated CRP levels are commonly associated with increased inflammation in various pathological conditions. In our study, the positive correlation suggests that as inflammation increases, the proportion of circulating megakaryocytes also increases.

Furthermore, our investigation revealed a potential mechanism by which CRP interacts with secreted protein acidic and rich in cysteine (SPARC) expressed by megakaryocytes to regulate virus response and immune regulation. SPARC is a multifunctional matricellular protein involved in various cellular processes, including immune modulation and tissue remodeling. The interaction between CRP and SPARC may affect the immune response and viral clearance in COVID-19.

The observed correlation between CRP and megakaryocytes, along with the potential interaction with SPARC, suggests that megakaryocytes could play a role in the immune response to COVID-19. Megakaryocytes are known to produce platelets, which have recently been implicated in immune regulation beyond their traditional role in hemostasis. The interaction between CRP and SPARC expressed by megakaryocytes may modulate the immune response and contribute to regulating viral clearance and inflammation in COVID-19 patients.

These findings highlight the complex interplay between inflammatory markers, megakaryocytes, and immune regulation in COVID-19. Understanding the mechanisms underlying the positive correlation between CRP and megakaryocytes, as well as the role of the CRP-SPARC interaction, could provide valuable insights into the pathogenesis of COVID-19 and potentially identify novel therapeutic targets.

It is essential to acknowledge the limitations of our study. Although we found a positive correlation between serum CRP levels and circulating megakaryocyte proportion, further investigations are needed to establish a causal relationship and elucidate the underlying mechanisms. Additionally, the generalizability of our findings may be influenced by factors such as patient population, disease severity, and comorbidities.

## Conclusion

Our study reveals a positive correlation between serum CRP levels and circulating megakaryocyte proportion, suggesting an interaction between CRP and megakaryocytes in COVID-19. Furthermore, the CRP-SPARC interaction may affect virus response and immune regulation. These findings contribute to understanding the intricate relationship between inflammation, megakaryocytes, and the immune response in COVID-19, offering potential avenues for further research and therapeutic interventions.

## Data availability statement

The original contributions presented in the study are included in the article/supplementary material. Further inquiries can be directed to the corresponding author.

## Ethics statement

The studies involving humans were approved by This study was reviewed by the Ethics Committee of Peking University Third Hospital (IRB00006761-M2020060). The studies were conducted in accordance with the local legislation and institutional requirements. The human samples used in this study were acquired from primarily isolated as part of your previous study for which ethical approval was obtained. Written informed consent for participation was not required from the participants or the participants’ legal guardians/next of kin in accordance with the national legislation and institutional requirements.

## Author contributions

CL: Conceptualization, Data curation, Formal Analysis, Writing – original draft, Writing – review & editing. CZ: Conceptualization, Data curation, Methodology, Writing – original draft. XS: Conceptualization, Data curation, Investigation, Methodology, Writing – review & editing. LL: Methodology, Investigation, Visualization, Writing – review & editing. QL: Conceptualization, Funding acquisition, Investigation, Writing – review & editing.
